# Evaluation of the antibacterial effect of titanium dioxide nanoparticles combined with acrylic laminates for functional orthodontic appliances: a randomized controlled clinical trial

**DOI:** 10.1186/s12903-023-03805-2

**Published:** 2024-01-04

**Authors:** Ghada M. Elabd, Waleed Eldars, Marwa S. Shamaa, Marwa A. Tawfik

**Affiliations:** 1 Department of Orthodontics, Faculty of Dentistry, Mansoura University, Mansoura, Egypt; 2 Department of Medical Microbiology and Immunology, Faculty of Medicine, Mansoura University, Mansoura, Egypt; 3 Department of Basic Medical Sciences, Faculty of Medicine, New Mansoura University, Mansoura, Egypt

**Keywords:** Antibacterial activity, Orthodontic functional appliances, Titanium dioxide nanoparticles

## Abstract

**Background:**

The objective of this study was to evaluate the antibacterial effect of titanium dioxide nanoparticles incorporated into the acrylic baseplates of the maxillary part of twin block appliances in orthodontic patients during the treatment period.

**Materials and methods:**

Twenty-six patients were selected randomly and divided into two groups(n = 13). Test group patients used orthodontic functional appliances containing 1% titanium dioxide nanoparticles in acrylic baseplates. Control group patients used orthodontic functional appliances without titanium dioxide nanoparticles in acrylic baseplates. Swap samples were taken from the palatal gingiva facing the fitting surface of the acrylic component of the maxillary part of a twin block appliance for each patient at five-time intervals (baseline sample, after one, two, four, and six months) and then cultured in blood agar plates to calculate bacterial colony count. The Mann‒Whitney U test and the Friedman test were used to compare data. Bonferroni correction (p value ≤ 0.05) was applied to detect significant differences.

**The results:**

showed a decrease in the bacterial colony count in the test group compared to the control group. Pairwise comparisons revealed a statistically significant difference in samples after four- and six-month groups (p values = 0.002 and 0.011, respectively) vs. the one-month test group. A higher statistically significant difference was observed in the six-month group (p-value = 0.037) vs. the baseline group in the control group.

**Conclusion:**

The addition of 1% titanium dioxide nanoparticles to acrylic baseplates of orthodontic functional appliances significantly reduced the bacterial colony count under the base plate after at least four months of application.

## Introduction

The orthodontic appliance acrylic baseplates are most frequently fabricated from polymethyl-methacrylate (PMMA) because of their ease of use and low cost [1]. Additionally, PMMAs are employed for early orthodontic appliances and personalized impression trays for individuals with cleft lip and palate.

Currently, Hawley retainers, removable and auxiliary fixed appliances, and routine orthodontic treatments frequently involve the use of PMMA resins [2]. Unfortunately, due to the long-term presence of orthodontic appliance baseplates in the mouth and the porosity of their surfaces, these orthodontic appliance baseplates can negatively affect the oral microbiota, encourage the growth of biofilms, and exacerbate periodontal disease, dental caries, and gingival inflammation [3]. It has been demonstrated that bacteria can enter these appliance acrylic bases as deeply as 1 to 2 mm, making disinfection challenging [4].

It is challenging due to the increase in the number of sites that retain plaque and poor mechanical plaque removal which is typically seen with orthodontic appliance baseplates [5].

Mechanical cleaning of appliance acrylic baseplates is beneficial for minimizing biofilm and microbial plaque buildup, especially when combined with antimicrobial treatments [6, 7]. However, because such measurements typically rely on patient compliance, they might not be the best option for children, elderly individuals, or people with disabilities. Therefore, it would be ideal to have an addition that significantly improves acrylic baseplate inhibitory actions while maintaining its biocompatibility [8].

Various PMMA modification techniques have been suggested to enhance the material’s antibacterial activity to address these issues [9]. By including substances with antifungal and antibacterial properties, researchers are constantly attempting to change the chemical composition of PMMA. The use of nanoparticles such as titanium dioxide (TiO2) has demonstrated abilities that prevent the growth of bacterial and fungal biofilms on the PMMA surface [10, 11].

The applications of nanoparticles (NPs) in biology and medicine are numerous. According to Kim et al., NPs and their ions can generate free radicals, which can induce oxidative stress that can permanently harm bacteria, including damaging their membrane, DNA, and mitochondria, leading to bacterial death [12].

TiO2 is approved for use as an additive in food and medicine. The Food and Drug Administration (FDA) Inactive Ingredients Guide for dental paste, oral capsules, suspensions, tablets, and dermal preparations in the United States includes it [13].

Due to its strong photoactivity, stability, and relatively low cost, TiO2 has been employed in a variety of industrial and environmental fields and has been considered a useful substance. It has been employed for decontamination and disinfection purposes, and water purification [14]. To achieve extra benefits, the particle sizes can be changed [15].

TiO2 nanoparticles exhibit good hardness, high corrosion resistance, and antibacterial activity. They are also nontoxic and chemically inert [16]. TiO2 has been proven to have an antibacterial effect by producing hydroxyl radicals that, when exposed to ultraviolet light, act against bacteria in an aqueous solution [17].

TiO2 nanoparticles have been applied in many fields of dentistry. It has been applied to PMMA scaffolds [18], orthodontic wires [19], and dental alginate [20]. It is also added to acrylic resin polymethyl methacrylate in completely edentulous patients [21], glass-ionomer, nickel-titanium and stainless steel archwires, and orthodontic composites [22–26]. A systematic review was performed to evaluate the effect of the addition of titanium dioxide nanoparticles on the antimicrobial properties of polymethyl methacrylate [27].

To test its antibacterial effect, titanium dioxide nanoparticles were added to the acrylic baseplates of the maxillary part of twin block appliances in orthodontic patients during the treatment period.

## **Material and methods**

### Ethical considerations

The Research Ethics Committee of the Faculty of Dentistry – Mansoura University, Mansoura, Egypt provided ethical permission under code No. (A04071221). Before the beginning of the treatment, each patient’s parents received information about the trial and provided their informed consent according to the guidelines of the Helsinki Declaration.

The trial was registered at https://register.clinicaltrials.gov/ with ID – NCT06051487 and first posted: on 25/09/2023.

### Sample size calculation

The sample size was calculated by using G*Power software (version 3.1.9.6) [28, 29].

Based on a literature review (Roghayeh Ghorbanzadeh et al. [30] and Farhadian et al. [31]), the authors hypothesize a medium effect size (f = 0.3) when using repeated-measures ANOVA (with in-between interaction) to compare bacterial count (log_10_ CFU/ml) between the two groups (test vs. control) over the 5-time points (at baseline, after 1_month, after 2_months, after 4_months, and after 6_months).

A total of 20 participants (10 in the test arm, and 10 as control subjects) achieved 92.7% power at a 5% significance level, 0.5 correlation among repeated measures, and one nonsphericity correction (ε = 1). Assuming a dropout rate of 20%, the enrollment sample size will be inflated to 13 participants per group.

### Participants, eligibility criteria, and settings

A total of 40 patients from the outpatient clinic of the orthodontic department, faculty of dentistry, Mansoura University, Mansoura, Egypt, were enrolled for orthodontic treatment. To ensure that the size of the groups was comparable, 26 individuals were randomly assigned to one of two groups after applying the inclusion and exclusion criteria. The flow chart for the Consolidated Standards of Reporting Trials (CONSORT) provided direction for the study’s design and presentation.

The inclusion criteria were as follows: early permanent dentition (ages 9 to 14), mandibular retrusion (SNB angle < 78°)-related class II skeletal deformity, overjet of more than 4 mm, bilateral class ll molar, and canine relation, and well aligned or minimal crowding in dental arches that can be aligned. Exclusion criteria were as follows: systemic diseases, congenital craniofacial deformity, inflammatory or infectious diseases within the previous month, taking medication in the last month, such as fluoride or antibiotics, frequent consumers of sorbitol- and xylitol-containing products, mouth rinses, unusual or specific dietary habits, cleft lip or palate, and unusual habits or previous orthodontic treatment.

### Trial design

A 1:1 parallel arm randomized controlled clinical trial was conducted. To achieve a group ratio of 1:1 (test group: _13, controls: _13), a total of 26 patients were needed for the investigation. Two participants did not continue using the appliance full-time, and four did not adhere to the instructions, resulting in the elimination of six persons from the trial. Patients were divided randomly into two groups. The test group used orthodontic functional appliances containing 1% titanium dioxide nanoparticles in acrylic baseplates. The control group used orthodontic functional appliances not containing titanium dioxide nanoparticles in acrylic baseplates. A twin block functional appliance used to treat patients with class II mandibular deficiency was the appliance used in this study.

### Randomization and blinding

**Fig. 1:**
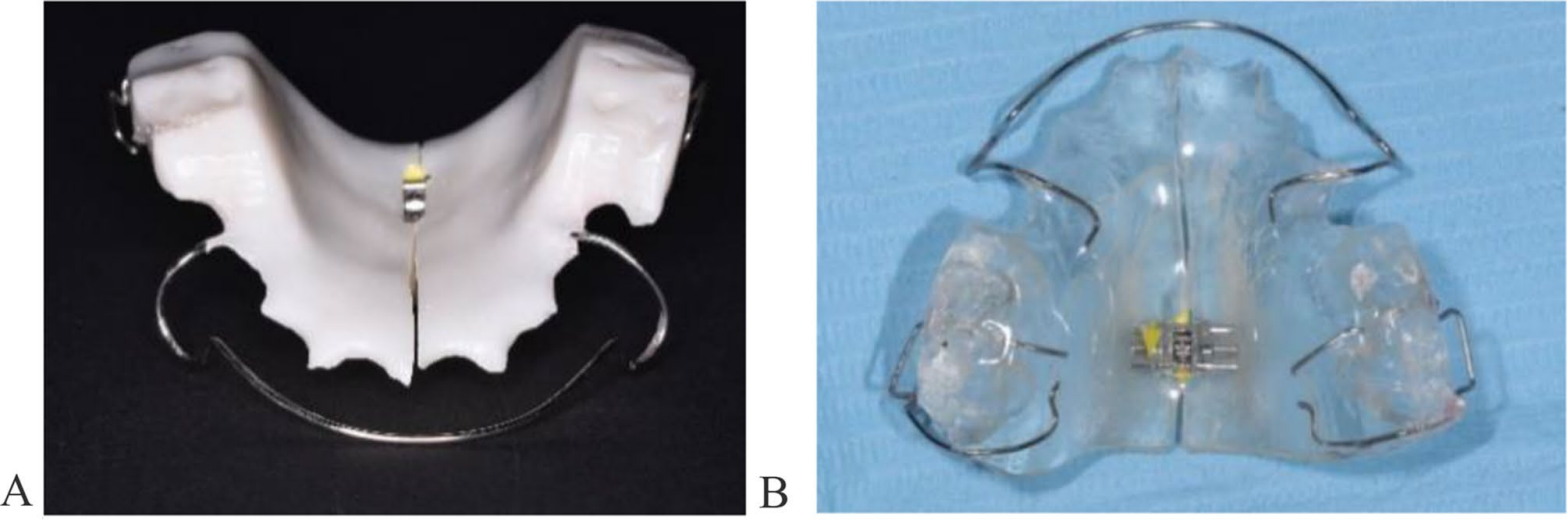
(**A**): Acrylic base plates containing titanium dioxide nanoparticles. (**B**) Acrylic base plates not containing titanium dioxide nanoparticles

Participants were coded and assigned numbers, which were subsequently divided into two equal groups (n = 13) using randomization software available at https://www.random.org/. The participants, the microbiologist and the laboratory technicians who constructed the appliances were blinded to the intervention. Patients were not able to distinguish between the two groups of twin block appliances based on color, but the investigators were able to do due to the whiter color of the baseplate of the twin block appliance containing 1% titanium dioxide nanoparticles (Fig. 1).

### Design of the appliance

For all of the chosen patients, the Twin Block, which consists of upper and lower bite blocks, was constructed. The upper part incorporates a labial bow with a U loop around the canines and Adam clasps on the upper first molars for retention. A midline screw was added to the upper part of all appliances to aid in the correction of the posterior crossbite if it occurred. The screw was not coated with Tio2 nanoparticles.

### Appliance construction (dental laboratory step)

Using the appropriate size orthodontic trays, an impression was made of the upper and lower arches using rubber base impression material. Using the Exacto bite, a construction wax bite was done. The mandible was positioned anteriorly to form an edge-to-edge relationship parallel to the functional occlusal plane. Improved plaster material was used to pour the impressions. the models were articulated with mandibular protrusion. Autopolymerizing PMMA resin was used to manufacture acrylic baseplates for orthodontic appliances based on cast models of each patient. Standard acrylization, trimming, and finishing-polishing processes were followed using the manufacturer’s recommended monomer-to-polymer ratio.

The acrylic material used for the fabrication of the appliance was (Acrostone acrylic material, under exclusive license, England). The manufacturer’s suggested powder-liquid volume ratio is 3:1 representing 22 g of the polymer and 10 mL of the liquid monomer. The powder-liquid volume ratio for the construction of the appliance was calculated (Table 1) [32].

**Table 1:** Composition of the acrylic baseplate

composition	PMMA (control)	PMMA (test)
TiO2 NP powder	0 g	1.12 g
PMMA powder polymer	77 g	77 g
Liquid monomer of PMMA	35 g	35 g

### Intervention

Titanium dioxide nanoparticles were prepared at the City of Scientific Research and Technological Applications (SRTA-City) in Egypt.

During the appliance containing 1% TiO2 nanoparticles construction, first, the calculated quantity of titanium dioxide nanopowder (Table 1) was mixed manually with acrylic resin polymer for 60 s by shaking the powders inside a small container and subsequently, the calculated quantity of the monomer was added to obtain a PMMA containing TiO_2_ NPs [32]. The powder was mixed with the liquid as recommended by the manufacturer, applied to a mold, and flasked. Then, the acrylic baseplate was finished and polished.

### Clinical step

Patients were instructed to use the device (twin block) full-time, including while they slept. The patients were instructed not to use mouthwashes, antibiotics, or xylitol products during this time but to continue brushing their teeth as usual and eating a normal diet instead. Before sample collection, the patients were told not to brush their teeth or practice other oral hygiene routines for 24 h, and they were also told not to eat or drink for an hour [30].

### Sample

Swab Samples were taken just before wearing the appliance (baseline sample) and then at the following time intervals of wearing the appliance for each group (after one month, after two months, after four months, and after six months).

**Fig. 2:**
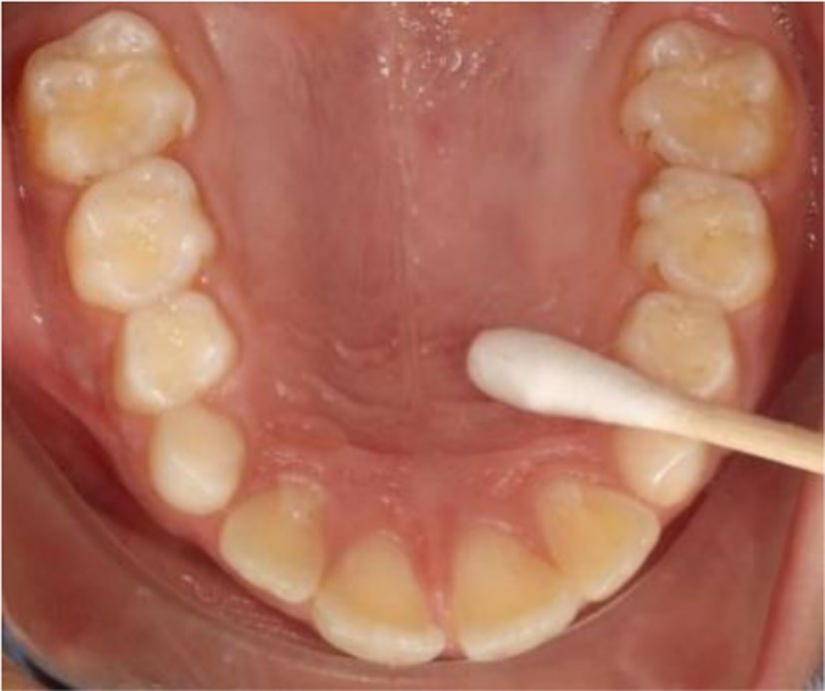
Swab from the palatal gingiva

**Fig. 3:**
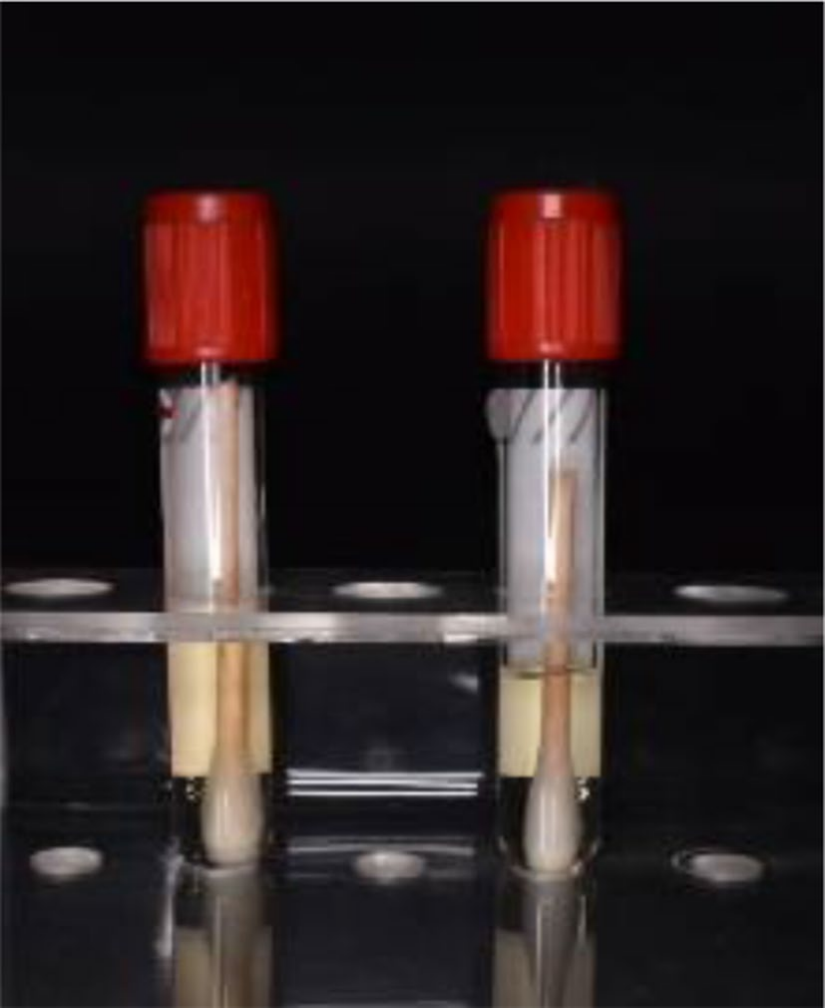
Sterilized tube containing broth solution and swap after taking the sample

The swab was taken from the palatal gingiva facing the fitting surface of the acrylic baseplate of the maxillary part of the twin block appliance for each patient using a sterilized cotton swab (Fig. 2) and then placed in 1 ml of broth solution in the sterilized tube (Fig. 3).

Broth solution (thioglycolate broth) which acts as transport media used to preserve bacteria if it is present until it is sent to the laboratory.

### Sample examination (laboratory step)

Sample examination was performed in the Microbiology Diagnostics and Infection Control Unit (MDICU), Medical Microbiology and Immunology Department, Faculty of Medicine, Mansoura University, Mansoura, Egypt.

**Fig. 4:**
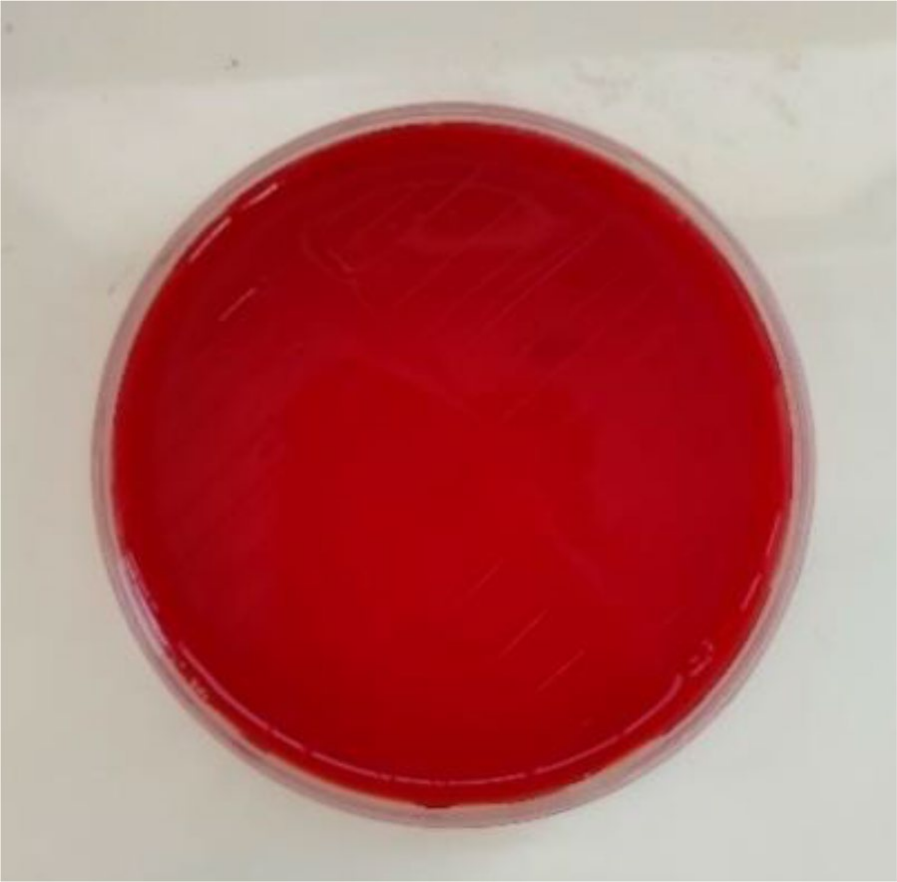
Blood agar plate

Samples were subjected to stirring using a Vortex Mixer VM-300 Gemmy for one or two minutes to release all samples from the swap in the solution. Furthermore, the broth solution was cultured in a blood agar plate (Fig. 4). Streptococci are generally grown on agar media supplemented with blood [33]. The solution was placed on the top of the plate using a swab and descended on a vertical line in the middle part of the plate. Then, horizontal lines passed through this vertical line, and the plate was placed in the incubator for 24 h to calculate the colony forming unit/ml for *streptococcus mutans*.

### Statistical analysis

IBM-SPSS software (IBM Corp. Released 2019. IBM SPSS Statistics for Windows, Version 26.0. Armonk, NY: IBM Corp) was used to enter and analyze the data. Qualitative data were expressed as N (%). Quantitative data were expressed as the mean ± SD if normally distributed or median and interquartile range (Q1 or 25th percentile – Q3 or 75th percentile) if not. To compare nonnormally distributed quantitative data between two groups, the Mann‒Whitney U test was used. The Friedman test was used to compare the repeatedly measured data in each group. For statistically significant differences, pairwise comparisons were performed, and significance values were adjusted by the Bonferroni correction for multiple tests. The results were considered statistically significant if the p value ≤ 0.05.

## Result

### Participant flow

**Fig. 5:**
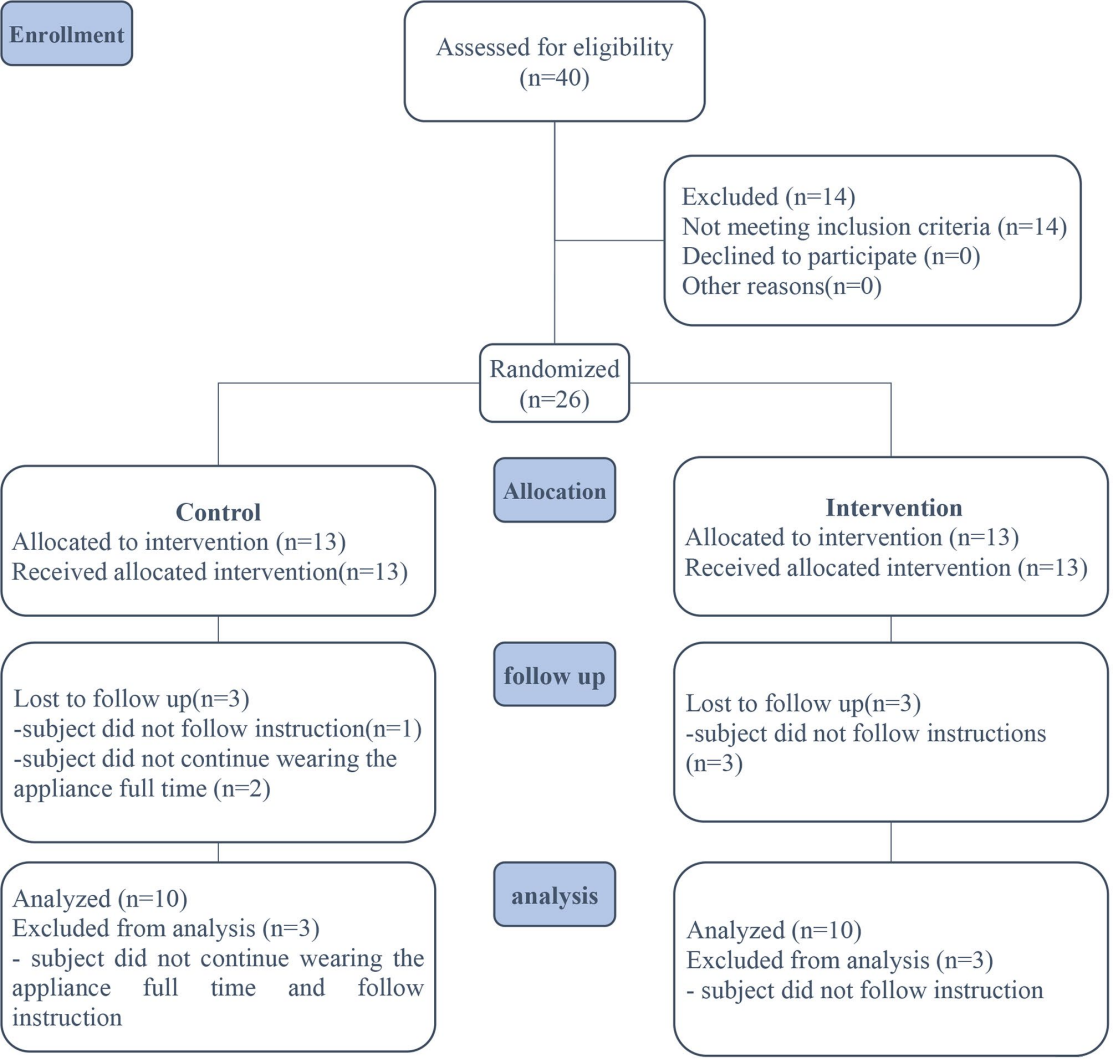
CONSORT flow diagram

The data were analyzed after excluding patients who were missing. Six participants (3 in each group) were removed from the trial because they did not cooperate. The final analysis of the antibacterial properties of titanium dioxide nanoparticles was assessed in 20 individuals (10 per group) who completed the research successfully (Fig. 5).

In this study, the patients were divided randomly into two groups. In the test group, there were 6 males and 4 females (mean age, 11 ± 1.16). In the control group, there were 5 males and 5 females (mean age, 10.6 ± 1.17). There were no significant differences in age or sex distribution between the test and control groups (Table 2).

**Table 2:** Age and sex distribution in the two groups

Characteristic	Test Group	Control group	Test of significance	
Sex Male Female	**N (%)** 6 (60%) 4 (40%)	**N (%)** 5 (50%) 5(50%)	**χ** ^**2**^ -	**P value** 1.000
Age (years)	**Mean ± SD** 11 ± 1.16	**Mean ± SD** 10.6 ± 1.17	**t value** 0.768	**P value** 0.452

*Notes*: N = absolute frequency. **χ**^**2**^ = chi-square. SD = standard deviation. The test of significance is Fisher’s exact test for sex and independent-samples t-test for age

**Fig. 6:**
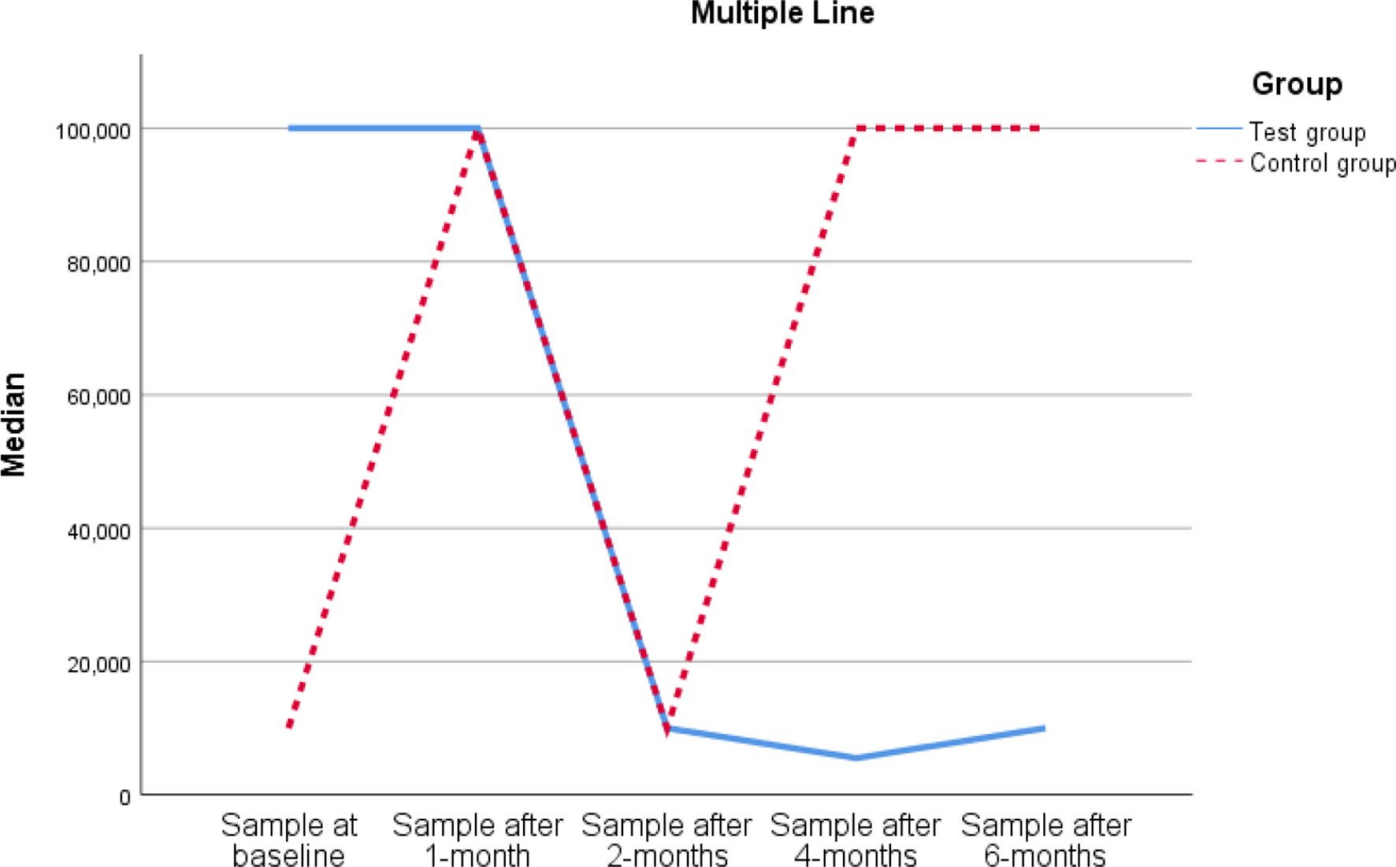
Multiple line graph for samples in both groups at each time point

On comparisons between the time points in each group:

A statistically significant difference in samples was shown between the 5 time points in each group (Table 3; Fig. 6). Pairwise comparisons revealed a statistically significantly lower sample after 4 and 6 months vs. 1 month in the test group (p values are 0.002 and 0.011, respectively) and a statistically significantly higher sample after 6 months vs. baseline in the control group (p = 0.037).

**Table 3:** Comparisons between the time points in each group

Time point	Test Group	χ^2^ [4]	P value	Control group	χ^2^ [4]	P value
At baseline	100,000 (100,000–100,000)	23.234	**< 0.001**	10,000 (1000–10,000)	15.465	**0.004**
After 1-month	100,000 (1,000,000 − 100,000)			100,000 (77,500–100,000)		
After 2-months	10,000 (7750-100000)			10,000 (10,000–100,000)		
After 4-months	5500 (775-10000)			100,000 (10,000–100,000)		
After 6-months	10,000 (1000–10,000)			100,000 (77,500–100,000)		

*Notes*: Data are median (Q1-Q3). The test of significance is the Friedman test

On comparison between the two groups at each time point:

At baseline, there were statistically significantly higher samples (p = 0.043), after 4 months, there were significantly fewer samples (p = **0**.005), and after 6 months, there were significantly fewer samples (p = 0.002) in the test group vs. the control group (Table 4).

**Table 4:** Comparisons between the two groups at each time point

Time point	Test Group	Control group	HLE	Z value	P value
At baseline	100,000 (100,000–100,000)	10,000 (1000–10,000)	90,000	-2.195	**0.043**
After 1-month	100,000 (1,000,000 − 100,000)	100,000 (77,500–100,000)	0.000	-0.669	0.684
After 2-months	10,000 (7750-100000)	10,000 (10,000–100,000)	0.000	0.935	0.436
After 4-months	5500 (775-10000)	100,000 (10,000–100,000)	-90,000	2.865	**0.005**
After 6-months	10,000 (1000–10,000)	100,000 (77,500–100,000)	-90,000	3.170	**0.002**

*Notes*: Data are median (Q1-Q3). HLE = Hodges-Lehman estimator of median difference. The test of significance is Mann‒Whitney U test

On comparisons of changes in samples over time between the two groups:

Changes from baseline to 4 months (baseline *minus* one month) showed a statistically significantly higher change in samples (p value < 0.001).

**Fig. 7:**
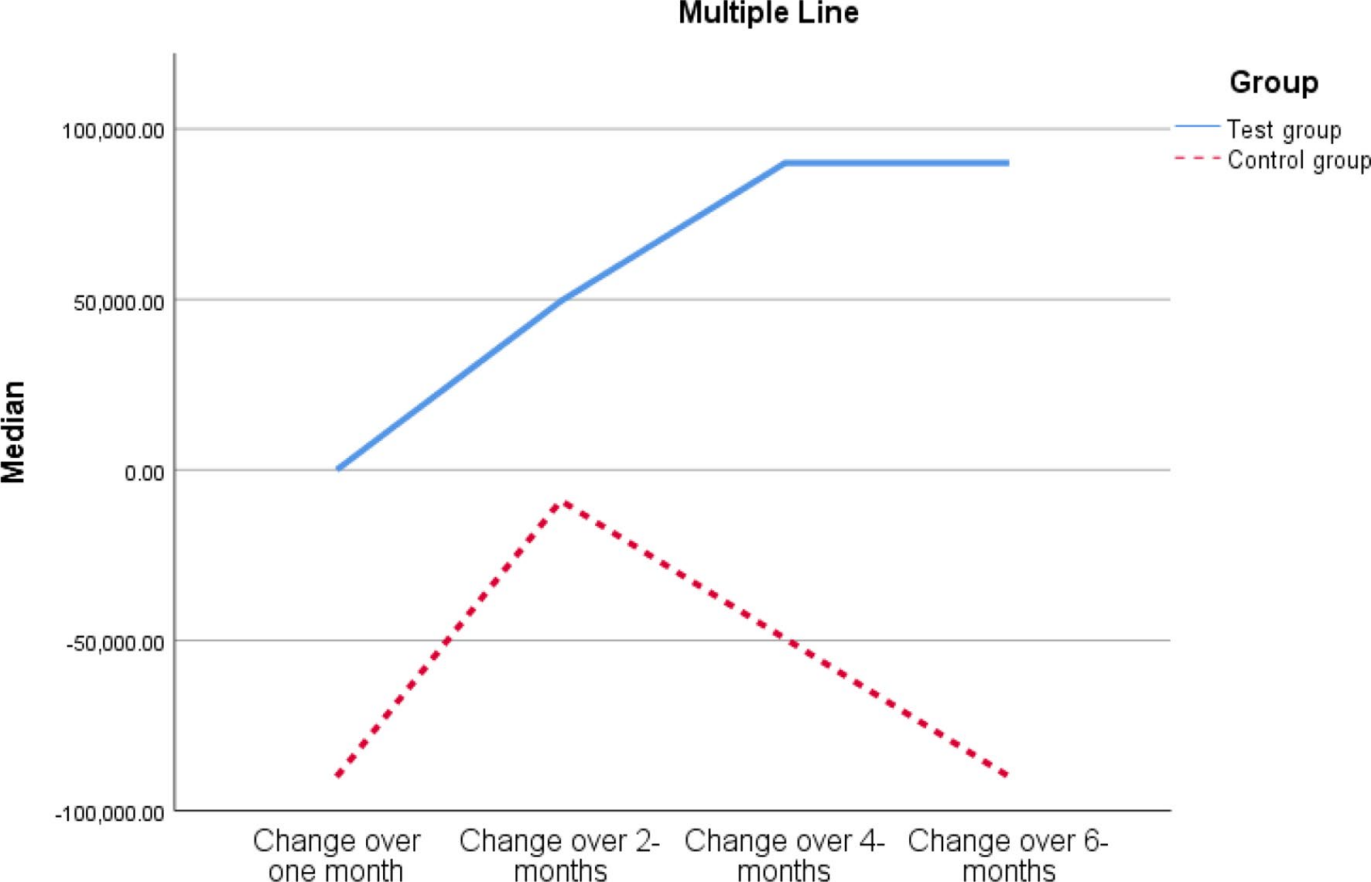
Multiple line graph for the changes in samples over time

Changes from baseline to 6 months (baseline *minus* six months) showed a statistically significantly higher change in samples (p value < 0.001) in the test group vs. the control group (Table 5; Fig. 7).

**Table 5:** Comparisons of changes in samples over time between the two groups

Change	Test Group	Control group	HLE	Z value	P value
Baseline *minus* one-month	0 -90,000 to 0	-90,000 -92,250 to 0	9900	-1.656	0.143
Baseline *minus* two months	49,500 -22,500 to 90,000	-9000 -90,000 to 0	90,000	-1.966	0.052
Baseline *minus* four months	90,000 6750 to 92,250	-49,500 -90,000 to 0	99,000	-3.608	**< 0.001**
Baseline *minus* six months	90,000 0 to 99,000	-90,000 -92,250 to -675	99,900	-3.453	**< 0.001**

*Notes*: Data are median, and Q1 to Q3. HLE = Hodges-Lehman estimator of median difference. The test of significance is Mann‒Whitney U test

### Harms

No participant displayed any symptoms of an allergic reaction or had complaints about taste or appearance.

## Discussion

Dental caries, gingival inflammation, and periodontal disease could be caused by the colonization and plaque production of bacteria on the baseplate of orthodontic appliances [32–35]. To address this issue, the use of PMMA that contains antimicrobial agents or coatings is advised [25–28]. Therefore, a broad-spectrum antimicrobial resin is needed to minimize side effects following treatment without the risk of developing resistant species of bacteria.

Orthodontic appliances that include nanoparticles or are coated with them may be able to prevent the formation of cariogenic bacteria. This study evaluated the antibacterial performance of titanium dioxide nanoparticles applied to the acrylic baseplate of a twin block orthodontic appliance, which is one of the most widely used functional appliances for treating skeletal class II disorders because it is well tolerated by patients and facilitates speaking and mastication [34, 35]. A combination of skeletal and dentoalveolar alterations made by the twin block appliance aids in treating the malocclusion. The compliance of the patients for wear of the twin block was good, as patients were motivated to improve their appearance, and follow-up of the patients was performed regularly to ensure wearing the appliance.

In this study, the upper part was used because it contains a larger surface area of the acrylic base plate covering the palate.

Due to their low toxicity on living cells when compared to other NPs, TiO2 NPs are thought to be the ideal candidate to be added to PMMA [36–39].TiO2 NPs show a broad range of antimicrobial action against bacteria, fungi, and both gram-negative and gram-positive bacteria [9]. No interaction occurs between TiO2 nanoparticles and the acrylic sheets of functional orthodontic appliances, as adding TiO2 nanoparticles to PMMA can improve its mechanical stiffness, wear resistance, and fracture resistance, and it can reduce its roughness [40].

Titanium dioxide nanoparticles are added to acrylic sheets used in functional orthodontic appliances at a concentration that does not affect the soft tissues of the mouth. There are no symptoms of an allergic reaction or complaints about the taste or appearance observed.

As the timing of treatment with twin block may run from 9 to 12 months, samples were obtained at various intervals up to 6 months. A baseline sample was obtained for comparison with the final sample.

A midline screw was added to the upper part of all the appliances. The screw was not coated with TiO2, as it was not opened during the study.

In this study, the results showed a statistically significantly lower bacterial colony count after 4 and 6 months vs. 1 month in the test group and a statistically significantly higher bacterial colony count after 6 months vs. at baseline in the control group.

During comparison between the two groups, the results showed a significantly higher bacterial colony count at baseline and a statistically significantly lower bacterial colony count after 4 and 6 months in the test group vs. the control group. TiO2 has been proven to have an antibacterial effect by producing hydroxyl radicals that, when exposed to ultraviolet light, act against bacteria in aqueous solution [16, 17].

This clinical trial showed that patients using twin block appliances made of an acrylic resin containing titanium dioxide nanoparticles had significantly fewer bacterial colony counts; this difference was statistically significant. Bacterial colony counts were higher in the control group with twin block appliances using conventional acrylic base plates, which coincides with a previous study that applied it on PMMA scaffolds [18] and dental alginate [20] and a systematic review evaluating the effect of the addition of titanium dioxide nanoparticles on the antimicrobial properties of polymethyl methacrylate [27]. This finding also coincides with previous clinical studies that have proven the efficacy of TiO_2_ as an antibacterial agent when applied on acrylic resin polymethyl methacrylate in completely edentulous patients [21], stainless steel and nickel-titanium archwires and coincides with a previous study that applied it on glass-ionomer and orthodontic composites [22–26].

The mechanical characteristics of acrylic resin were not examined in this investigation, and the main objective was to focus on the antimicrobial properties; however, unusual breakage of the twin block appliance was not observed during the study. Loghman Ghahremani et al. [41] concluded that a color-modified acrylic resin supplemented with TiO2 had noticeably higher tensile and impact strength than the conventional acrylic resin.

## Conclusion

Adding 1% titanium dioxide nanoparticles to the acrylic baseplate of an orthodontic functional appliance reduces bacterial colony count under the acrylic baseplate after at least four months of application.

## Data Availability

The collected and analyzed datasets are available from the corresponding author upon reasonable request.
